# Socioeconomic Status and Stroke Prevalence in Morocco: Results from the Rabat-Casablanca Study

**DOI:** 10.1371/journal.pone.0089271

**Published:** 2014-02-28

**Authors:** Thomas Engels, Quentin Baglione, Martine Audibert, Anne Viallefont, Fouzi Mourji, Mustapha El Alaoui Faris, for the GRAVCM Study Group

**Affiliations:** 1 CERDI-CNRS, University of Auvergne, Clermont-Ferrand, France; 2 Hassan II Aïn Chock University and LASAARE, Casablanca, Morocco; 3 Department of Neurology and Neuropsychology, Hôpital des Spécialités and Mohamed V-Souissi University, Rabat, Morocco; University Medical Center Rotterdam, Netherlands

## Abstract

**Background:**

Stroke is a growing public health concern in low- and middle- income countries. Improved knowledge about the association between socioeconomic status and stroke in these countries would enable the development of effective stroke prevention and management strategies. This study presents the association between socioeconomic status and the prevalence of stroke in Morocco, a lower middle-income country.

**Methods:**

Data on the prevalence of stroke and stroke-related risk factors were collected during a large population-based survey. The diagnosis of stroke in surviving patients was confirmed by neurologists while health, demographic, and socioeconomic characteristics of households were collected using structured questionnaires. We used Multiple Correspondence Analysis to develop a wealth index based on characteristics of the household dwelling as well as ownership of selected assets. We used logistic regressions controlling for multiple variables to assess the statistical association between socioeconomic status and stroke.

**Findings:**

Our results showed a significant association between household socioeconomic status and the prevalence of stroke. This relationship was non-linear, with individuals from both the poorest (mainly rural) and richest (mainly urban) households having a lower prevalence of stroke as compared to individuals with medium wealth level. The latter belonged mainly to urban households with a lower socioeconomic status. When taking into account the urban population only, we observed that a third of poorest households experienced a significantly higher prevalence of stroke compared to the richest third (OR = 2.06; CI 95%: 1.09; 3.89).

**Conclusion:**

We conclude that individuals from the most deprived urban households bear a higher risk of stroke than the rest of the population in Morocco. This result can be explained to a certain extent by the higher presence of behavioral risk factors in this specific category of the population, which leads in turn to metabolic and physiological risk factors of stroke, such as obesity, diabetes, and hypertension.

## Background

### Global burden of stroke

The World Health Organization (WHO) estimates that a total of 57 million deaths occurred worldwide in 2008, of which 36 million were due to non-communicable diseases. Among these, cerebrovascular disease (stroke) is one of the leading causes of mortality with an estimated 6.2 million deaths, representing nearly 11% of all global deaths [1]–[2].

Stroke was ranked as the seventh leading cause of disability-adjusted life-years (DALYs) lost in 2002, and projections indicate that it will be the second leading cause of deaths, and the sixth cause of DALYs lost globally by 2030 [3].

### Stroke epidemics in low- and middle- income countries

As demographic and disease transitions take place in developing countries, stroke is becoming a major health problem in low-income and middle-income countries (LMICs).

The projected increase in mortality due to stroke is expected to be faster in LMICs than in high-income countries (HICs), as a result of the increasing prevalence of risk factors (due to both an ageing population and changes in lifestyle) and lesser availability of primary prevention and acute care programs [4]–[7]. A systematic review of population-based studies carried out from 1970–2008 showed a 42% decrease in incidence of stroke in high-income countries, as compared to a more than 100% increase in incidence in LMICs [6], [8]. Recently, epidemiological studies in countries such as India, Chile and South Africa, have shown similar incidence of stroke as in developed countries; yet the mortality due to stroke remains higher in LMICs [7].

By 2030, stroke is projected to be both the first leading cause of death (14.4% of total deaths) and the third leading cause of DALYs lost (6% of total DALYs) in middle income countries; and the third leading cause of death (8,2% of total deaths) and the 8^th^ leading cause of DALYs lost (2.8% of total DALYs) in low-income countries [3]. Without effective preventive measures to curb this trend, the economic impact of such a stark increase in burden due to stroke could be devastating to future global economic growth [9].

### Stroke and socioeconomic status

Differences in burden due to stroke also exist within countries where the incidence and mortality rates vary across socioeconomic groups. A substantial number of studies in western countries have demonstrated a significant positive association between socioeconomic disadvantage and the incidence and mortality due to stroke [10], [11]. By contrast, the few studies available in developing countries show various patterns as the social gradient for risk factors leading to stroke may change over time. Recent studies investigating such patterns in transitioning economies have supported the fact that risky behaviors associated with stroke and other cardio-vascular diseases are initially higher within the highest socioeconomic classes (early adopters), but the socioeconomic gradient is gradually inversed and with time the burden increasingly supported by the poor [12]–[15]. The same inversion of the social gradient was observed for HICs throughout their past evolution [10]. This social transition occurs because those of high socioeconomic status (SES), who were early adopters of poor health behaviors (such as smoking and diets rich in processed foods with more fat, sugar, and salt), are also the first to respond to health messages and recognize more rapidly that their lifestyles are not conducive to a healthy life. Those of higher SES also have the resources to change their behaviors and environment to decrease their risk [11].

Most research and attention to stroke prevention and intervention measures occurs in high-income countries, while more than 85% of the cases of stroke occur in low-income and middle-income countries [6]. Knowledge about disparities in risk factors is important in order to define targeted and effective stroke prevention and management strategies [7]. This study examines the association between SES and the prevalence of stroke in Morocco, a lower middle-income country according to the World Bank categorization of countries by income groups. Morocco is currently going through substantial nutritional, epidemiological and demographic transitions. As a result, the prevalence of risk factors associated with cardiovascular disease is increasing and stroke is a growing public health concern [16]–[20]. Several studies have investigated the link between socioeconomic status and health in Morocco, highlighting strong social and health inequities among rural and urban populations and wealth income groups [21]–[24].

## Methods

### Ethics statement

The ethics committee of the Faculty of Medicine and Pharmacy of Rabat (Morocco) has specifically approved this study. Participants provided verbal informed consent to participate in the survey after a note in dialectal Arabic was read out loud to present the study. The use of oral consent instead of written consent was approved by the ethics committee considering the low level of literacy in the studied population. No formal documentation of households refusing to take part in the study was kept. We recorded that 3% of households declined to participate in the study for a variety of reasons including lack of time or interest that were not specifically related to the content of the study.

### Data

This study is based on data collected during a large-scale population-based survey in the regions of Casablanca and Rabat between November 2008 and April 2009.

A stratified random sampling method was adopted using Primary Units established by the *Haut Commissariat au Plan* [High Commission for Planning] based on the 2004 general population census. Data from 13,279 households (60,031 individuals) were collected by questionnaire during a door-to-door survey. Comparison of the sampled population with the overall Moroccan population indicates that the sample is representative with regards to demographic and socioeconomic criteria [25].

The survey was conducted in two consecutive steps. In the first phase, household characteristics were collected and a preliminary screening for history of stroke was performed through a questionnaire by 34 trained interviewers and 6 supervisors.

The first part of the questionnaire focused on the composition and socio-demographic characteristics of households. Age, gender, marital status, occupation and education were assessed for each household member. To be considered as a member of the household, an individual had to share at least one meal a day for minimum six months prior to the date of the interview.

The second part included questions related to health and lifestyle habits (such as diet, smoking, and sport). History of chronic diseases and health seeking behaviors were assessed for each household member. Diagnosis of hypertension; diabetes, heart diseases, neuropsychiatric disorders, respiratory diseases, and rheumatism were self-reported. History of diabetes and hypertension were only recorded if confirmed by a doctor prior to the time of the interview. Cardiac diseases included the following conditions: Ischemic heart disease, rheumatic heart disease and hypertensive heart disease. The diagnosis was confirmed by reviewing data from cardiologist examination, electrocardiography, and echography. Neuropsychiatric disorders refer to history of psychiatric disorders before the onset of stroke, usually depression or anxiety disorders requiring psychiatrist's consultation. The category “other diseases” captured all chronic diseases that were not considered in the above categories such as kidney disorders or arteriopathy of the lower limb. Questions on health seeking behaviors included frequency and type of consultations. The type and frequency of sport practice was also recorded. No threshold was used for defining smoker status but questions were included to assess years of smoking and the number of cigarettes smoked per day. Diet characteristics were assessed for each household by asking questions on frequency of consumption of specific types of food.

The third part of the questionnaire focused on housing characteristics for each household, including construction materials, availability of electricity, water and sanitation facilities. Data were also collected on ownership of various durable goods, such as television, refrigerator, car and telephone.

The last part consisted in a screening questionnaire to detect potential stroke. It aimed at identifying probable stroke patients as well as households in which a person had possibly died of stroke.

In a second phase, alleged cases of stroke were further investigated by a team of 18 neurologists in order to either confirm the diagnosis of stroke in surviving patients or to confirm stroke as a cause of death.

Strokes were diagnosed following WHO standard stroke definition: A focal (or at times global) neurological impairment of sudden onset, and lasting more than 24 hours (or leading to death), and of presumed vascular origin [26]. Neurologists examined all suspected stroke survivors, recording the history of stroke and risk factors including hypertension, diabetes, atrial fibrillation and smoking. They also performed a neurological and cardiac examination, and checked data of brain Computed Tomography (CT) scan or Magnetic Resonance Imaging (MRI). Functional status was assessed by using modified Rankin and Barthel indexes and Instrumental Activities of Daily Living (IADL). Finally, cognitive status and depressive symptoms were evaluated using two neurobehavioral tests, namely the Mini-Mental State Examination (MMSE) and the Montgomery-Åsberg Depression Rating Scale (MADRS).

### Construction of a wealth index

Material assets or wealth index can be a valuable measure of socioeconomic status, similar to other commonly used measures, such as occupation, educational level or income [27]–[29]. This approach is a particularly useful alternative to measuring households income and consumption expenditure in developing countries [27], [30].

Multiple Correspondence Analysis (MCA) was used to construct a wealth index based on 15 variables: type of housing, number of rooms, type of lighting, source of water supply, presence of housing amenities (kitchen, bathroom and toilet), and the household's possession of durable assets (television, satellite dish, phone and cell phone, stove, fridge, washing machine, and car). Details regarding the value labels, codes and frequencies of each modality of the 15 variables considered for the MCA are provided in table S1.

The MCA method consists in summarizing several categorical variables related to the dwelling and ownership of durable goods into fewer quantitative variables or indices. We used only the first factorial axis for our analysis since it represents 79.4% of the total variability. Given that the first factorial axis explains a great proportion of the variance (information), it is regarded as a good synthetic indicator of the overall housing characteristics and can therefore be used as a proxy for the household economic status.

The value of the wealth index ranged from −2.92 to 2.04 but such raw scores are fairly meaningless when taken individually. Nevertheless, such a score is appropriate for statistical analysis and to rank individuals according to the index value for the household they belong to. Households with the highest score are the wealthiest and are comparatively better off than households with lower scores. It is also possible to use the index to group individuals into a limited number of categories e.g. population tertiles, from 1 (poorest) to 3 (wealthiest).

### Multiple logit regressions

We examined the statistical link between SES and the prevalence of stroke using logistic regressions. We tested the effect of the wealth index previously computed and other predictive variables: sex, age and a set of behavioral or metabolic risk factors. We then progressively dropped from the model the few non-significant control variables, in order to ensure a good statistical power for testing the link between the variable of interest, Index, and the history of stroke. The variables that were dropped from the model include: education, occupation, tobacco smoking, practice of sport, and respiratory disorders.

First, we examined the contribution of SES to the prevalence of stroke in the population aged 15 years or older (N = 44,742) by introducing the wealth index (Index) and its quadratic form (Index^2^), expressed as continuous variables. We also examined the contribution of SES to the prevalence of stroke in urban (N = 28,179) and rural areas (N = 16,563) separately. Morocco is indeed characterized by a sharp contrast between urban and rural areas in terms of life style, education, poverty and access to healthcare [21], [31]. Such contrasting lifestyles are typical for countries in early and mid-transition phases [32].

Secondly, we examined the contribution of SES to the prevalence of stroke by classifying the urban population in 3 categories (poor, intermediate and rich individuals). The same was done for the rural population. We opted for a breakdown in tertiles instead of the more classical approach using quintiles in order to highlight substantial variations in the prevalence of stroke between the poorest (T1) and the richest (T3) in each sub-set of the population (i.e. urban and rural). We justify our choice by the fact that the results of our analysis are not altered by a breakdown in quintiles, yet interpretation of the results is more straightforward in terms of health policy recommendations.

Finally, we carried out chi-squared and t-tests to identify which first-level risk factors (such as health care utilization or smoking) tend to be more frequently associated to higher prevalence of stroke in the urban and rural populations. These behavioral factors are likely to participate indirectly in the risk of stroke through their effect on the metabolic risk factors used in the logistic regression analyses (i.e. hypertension, diabetes, etc.).

All statistical analysis were carried out using STATA 11.0 [StataCorp. 2009. Stata Statistical Software: Release 11. College Station, TX: StataCorp LP]

## Results

Table 1 provides information on baseline characteristics of the study population and history of stroke.

**Table 1:** Baseline characteristics of study population (15 and above) and history of stroke, prevalence by urban and rural areas (N = 44,742).

Characteristics	Population (n, %^a^)					
	study population, above 15 (N = 44,742)				Rural, above 15 (N = 16,563)	Urban, above 15 (N = 28,179)
	(n, %^a^)	Stroke (n, %^a^)	No stroke (n, %^a^)	P-value^b^	(n, %^a^)	(n, %^a^)
**Stroke**				-		
No	44,615 (0.99)	-	44,615 (0.9972)		16,507 (0.9966)	28,108 (0.9975)
Yes	127 (0.0028)	127 (0.0028)			56 (0.0034)	71 (0.0025)
**Age (years)**				0.000		
15–24	12,149 (27.1)	4 (3.1)	12,145 (27.2)		4,968 (30.0)	7,181 (25.5)
25–34	10,144 (22.7)	2 (1.6)	10,142 (22.7)		3,667 (22.1)	6,477 (23.0)
35–44	8,447 (18.9)	2 (1.6)	8,445 (18.9)		2,936 (17.7)	5,511 (19.6)
45–54	6,737 (15.1)	16 (12.6)	6,721 (15.1)		2,252 (13.6)	4,485 (15.9)
55–64	3,985 (8.9)	21 (16.5)	3,964 (8.9)		1,339 (8.1)	2,646 (9.4)
65+	3,280 (7.3)	82 (64.6)	3,198 (7.2)		1,401 (8.5)	1,879 (6.7)
**Gender**				0.365		
Male	21,808 (48.7)	67 (52.8)	21,741 (48.7)		8,249 (49.8)	13.559 (48.1)
Female	22,934 (51.3)	60 (47.2)	22,874 (51.3)		8,314 (50.2)	14,620 (51.9)
**Education (years)^c^**				0.000		
0–4	20,064 (44.8)	104 (81.9)	19,960 (44.7)		10,682 (64.5)	9,382 (33.3)
5–9	13,769 (30.8)	13 (10.2)	13,756 (30.8)		4,390 (26.5)	9,379 (33.3)
10+	10,909 (24.4)	10 (7.9)	10,899 (24.4)		1,491 (9.0)	9,418 (33.4)
**Cigarette smoking**				0.052		
No	39,802 (89.0)	118 (94.4)	39,684 (89.0)		14,756 (89.1)	25,046 (88.9)
Yes	4,932 (11.0)	7 (5.6)	4,925 (11.0)		1,806 (10.9)	3,216 (11.1)
Missing values	8	2	6		1	7
**Practice of sport**				0.001		
No	38,850 (86.8)	121 (96.8)	38,729 (86.8)		15,700 (94.8)	23,150 (82.2)
Yes	5,886 (13.2)	4 (3.2)	5,882 (13.2)		862 (5.2)	5,024 (17.8)
Missing values	6	2	4		1	5
**Hypertension**				0.000		
No	41,930 (93.7)	72 (56.7)	41,858 (93.8)		15,633 (94.4)	26,297 (93.3)
Yes	2,812 (6.3)	55 (43.3)	2,757 (6.2)		930 (5.6)	1,882 (6.7)
**Diabetes Mellitus**				0.000		
No	42,988 (96.1)	104 (81.9)	42,884 (96.1)		16,173 (97.7)	26,815 (96.2)
Yes	1,754 (3.9)	23 (18.1)	1,731 (3.9)		390 (2.3)	1,364 (4.8)
**Heart diseases**				0.000		
No	43,630 (97.5)	100 (78.7)	43,530 (97.6)		16,140 (97.5)	27,490 (97.5)
Yes	1,112 (2.5)	27 (21.3)	1,085 (2.4)		423 (2.5)	689 (2.5)
**Neuropsychiatric disor.**				0.000		
No	43,268 (96.7)	111 (87.4)	43,157 (96.7)		15,760 (95.1)	27,508 (97.6)
Yes	1,474 (3.3)	16 (12.6)	1,458 (3.3)		803 (4.9)	671 (2.4)
**Respiratory disorders**				0.785		
No	43,179 (96.5)	122 (96.0)	43,057 (96.5)		16,016 (96.7)	27,163 (96.4)
Yes	1,563 (3.5)	5 (3.9)	1,558 (3.5)		547 (3.3)	1,016 (3.6)
**Rheumatism**				0.988		
No	42,615 (95.3)	121 (95.3)	42,494 (95.3)		15,491 (93.5)	27,124 (96.3)
Yes	2,127 (4.7)	6 (4.7)	2,121 (4.7)		1,072 (6.5)	1,055 (3.7)
**Other diseases**				0.000		
No	41,546 (92.9)	66 (52.0)	41,480 (93.0)		15,037 (90.9)	26,509 (94.1)
Yes	3,196 (7.1)	61 (48.0)	3,135 (7.0)		1,526 (9.2)	1,670 (5.9)
**Nb of consultations^d^**				0.000		
0	27,115 (61.9)	38 (29.9)	27,077 (62.0)		10,484 (66.1)	16,631 (59.5)
1–3	11,651 (26.6)	30 (23.6)	11,621 (26.6)		3,732 (23.5)	7,919 (28.3)
4+	5,058 (11.5)	59 (46.5)	4,999 (11.4)		1,649 (10.4)	3,409 (12.2)
Missing values	918	0	918		698	220

Notes:

a n: number of participants within sub-groups; %: percentages across column.

b P-value between sub-groups of each variable.

c Number of years of schooling successfully completed.

d Number of consultations in the last 12 months, all type of healthcare providers considered.

A total of 127 cases of stroke were diagnosed, 56 cases were observed in rural areas and 71 in urban areas; leading to a crude prevalence of stroke of 284/100,000 (338/100,000 for rural areas and 252/100,000 for urban areas); when considering the population aged 15 and over. Stroke cases were classified in three major subtypes as follows: ischemic stroke (70.9%), intracerebral haemorrhage (8.7%), and subarachnoid haemorrhage (3.9%); 16.5% were undetermined.

**Figure 1:**
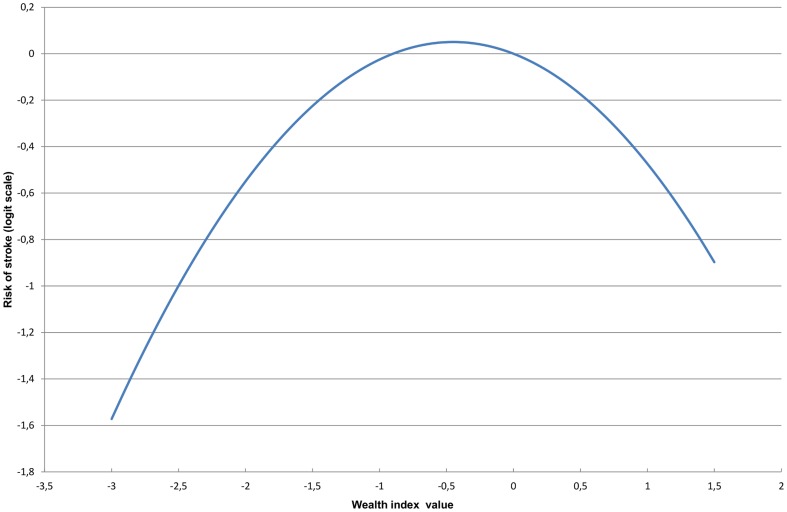
Relationship between SES and occurrence of stroke in study population aged over 15yrs (N = 44,742). The estimated probability of stroke is presented here on a logit scale and was obtained for different values of the wealth index after controlling for the following factors: Age, gender, and history of chronic diseases prior to stroke (hypertension, diabetes, heart diseases, neuropsychiatric disorders, rheumatism and other chronic diseases).

Our logit model indicates that in the study population aged over 15 years (N = 44,742), the socioeconomic status has an impact on the prevalence of stroke and that this relationship is non-linear; as shown in Figure 1. When the estimated probability of stroke is plotted for each value of the index, a concave function is obtained, indicating a higher occurrence for individuals with intermediate wealth indices. Whereas the coefficient associated with the variable Index is only significant at 10% (P-value  =  0.064), the coefficient related to Index^2^ is highly significant (P-value  =  0.012), and the likelihood-ratio test indicates that the model with “Index+ Index^2^” better fits the data than the model without these two factors (P-value  =  0.028).

Table 2 presents details of this relationship, as well as the association of diagnosed stroke with conventional risk factors: wealth index, age, gender and a set of pre-existing chronic conditions.

**Table 2:** Association of SES and history of stroke in study population aged over 15yrs (N = 44,742), wealth index introduced as a quantitative variable (Model 1).

Variables	Total population aged 15+			Rural population aged +15yrs			Urban population aged +15yrs		
	Coeff (95% CI)	Adj. OR (95% CI)^a^	P-value	Coeff (95% CI) a	Adj. OR (95% CI)	P-value	Coeff (95% CI)	Adj. OR (95% CI)^a^	P-value
**Index**	−0.22 (−0.46;0.013)		0.064^*^	−0.14 (−0.82;0.53)		0.678	−0.42 (−0.80;−0.05)		0.026^**^
**Index^2^**	−0.25 (−0.44;−0.054)		0.012^**^	−0.23 (−0.61;0.15)		0.238	−0.009 (−0.401;0.38)		0.961
**Age (year)**	0.18 (0.099;0.263)		0.000^***^	0.21 (0.08;0.33)		0.001^***^	0.13 (0.03;0.25)		0.011^**^
**Age^2^**	−0.0008 (−0.0015;−0.0002)		0.006^***^	−0.001 (−0.002;−0.0002)		0.015^**^	−0.0004 (−0.001;0.0004)		0.262
**Sex**									
Male		1			1			1	
Female		0.67 (0.47;0.98)	0.037^**^		0.46 (0.26;0.83)	0.009^***^		0.91 (0.55;1.49)	0.706
**Hypertension**									
No		1			1			1	
Yes		3.70 (2.48;5.51)	0.000^***^		5.34 (2.87;9.94)	0.000^***^		2.97 (1.75;5.02)	0.000^***^
**Diabetes Mellitus**									
No		1			1			1	
Yes		1.73 (1.06;2.84)	0.030^**^		0.91 (0.30;2.72)	0.866		2.08 (1.17;3.69)	0.012^**^
**Heart diseases**									
No		1			1			1	
Yes		2.99 (1.86;4.80)	0.000^***^		3.41 (1.63;7.14)	0.001^***^		2.70 (1.44;5.04)	0.002^***^
**Neuropsychiatric disorders**									
No		1			1			1	
Yes		2.96 (1.68;5.22)	0.000^***^		2.37 (1.08;5.22)	0.031^**^		3.74 (1.62;8.62)	0.002^***^
**Rheumatism**									
No		1			1			1	
Yes		0.26 (0.11;0.62)	0.002^***^		0.27 (0.09;0.79)	0.016^**^		0.25 (0.06;1.02)	0.054^*^
**Other diseases**									
No		1			1			1	
Yes		8.99 (6.13;13.18)	0.000^***^		10.5 (5.84;18.99)	0.000^***^		7.66 (4.58;12.82)	0.000^***^
**Intercept**	−13.23 (−15.91;−10.72)		0.000^***^	−13.72 (−17.62;−9.83)		0.000^***^	−12.62 (−16.06;−9.18)		0.000^***^
Correctly classified	85.03%			83.98%			86.25%		
Goodness of fit test^b^	0.169			0.726			0.707		
Pseudo R^2^	0.309			0.326			0.307		

Notes: *** significant at 1%; ** significant at 5%; * significant at 10%.

Coeff.: Coefficient; OR: Odds Ratio; CI: Confidence interval.

a Odds ratios adjusted for wealth index, age, gender, history of hypertension, diabetes, heart disease, neuropsychiatric disorders, rheumatism and other chronic diseases (prior to stroke).

b P-value provided for Hosmer-Lemeshow test.

The coefficient of Index^2^ is negative which indicates that the relation is concave with a lower prevalence for low and high SES households and a higher prevalence for intermediate SES households. Our results further indicate that the prevalence of stroke increases with age, and this relation is slightly concave. In terms of OR, women had a lower risk of stroke than men (OR = 0.67, 95% CI = 0.47, 0.98).The presence of high blood pressure (BP), diabetes, pre-existing heart and neuropsychiatric disorders increases the prevalence of stroke. The OR for stroke among subjects living with hypertension was 3.70 (95%CI = 2.48, 5.51) as compared with their counterparts without hypertension. The equivalent ORs for individuals with history of heart diseases, neuropsychiatric disorders, diabetes and other chronic diseases were 2.99 (95% CI = 1.86, 4.80), 2.96 (95% CI = 1.68, 5.22), 1.73 (95% CI = 1.06, 2.84) and 8.99 (95% CI = 6.13, 13.18), respectively. On the other hand, history of chronic rheumatism showed to be negatively correlated with the prevalence of stroke, with an OR of 0.26 (95% CI = 0.11, 0.62).

Our model does not show a significant association between stroke prevalence and the area of residence when the variable urban/rural is included in the regression (for the entire sample). However, when we look at the effect of the wealth index on rural and urban households separately, we observe that the coefficient of Index is negative and significant (P-value = 0.026) for urban residents (see table 2). This result suggests an inverse relation between socioeconomic status and prevalence of stroke in urban areas.

These findings were confirmed when repeating the analysis per tertile: the poorest households experienced a significant two-fold higher prevalence of stroke compared to the richest ones (OR = 2.06; CI 95%: 1.09; 3.89) when controlling for other confounding factors. The difference was not significant among rural households nor when looking at the population as a whole. These results are summarized in table 3.

**Table 3:** Association of SES and history of stroke in study population aged over 15yrs (N = 44,742), wealth index introduced in tertiles (Model 2).

Variables	Total population aged 15+			Rural population aged +15yrs			Urban population aged +15yrs		
	Coeff (95% CI)	Adj. OR (95% CI)^a^	P-value	Coeff (95% CI)	Adj. OR (95% CI)^a^	P-value	Coeff (95% CI)	Adj. OR (95% CI)^a^	P-value
**Index**									
Tertile 1 (Rich)		1			1			1	
Tertile 2 (Intermediate)		1.35 (0.84;2.17)	0.212		1.06 (0.56;1.99)	0.860		1.84 (0.98;3.44)	0.057^*^
Tertile 3 (Poor)		1.47 (0.92;2.32)	0.104		0.54 (0.26;1.13)	0.104		2.06 (1.09;3.89)	0.025^**^
**Age (year)**	0.18 (0.10;0.26)		0.000^***^	0.209 (0.086;0.332)		0.001^***^	0.14 (0.032;0.25)		0.011^**^
**Age^2^**	−0.0009 (−0.0015;−0.0002)		0.005^***^	−0.001 (−0.002;−0.0002)		0.015^**^	−0.0005 (−0.001;0.0004)		0.265
**Sex**									
Male		1			1			1	
Female		0.67 (0.46;0.97)	0.035^**^		0.46 (0.26;0.83)	0.010^**^		0.91 (0.56;1.49)	0.721
**Hypertension**									
No		1			1			1	
Yes		3.82 (2.56;5.69)	0.000^***^		5.28 (2.83;9.85)	0.000^***^		2.98 (1.76;5.04)	0.000^***^
**Diabetes Mellitus**									
No		1			1			1	
Yes		1.76 (1.07;2.88)	0.026^**^		0.89 (0.30;2.67)	0.838		2.05 (1.16;3.63)	0.014^**^
**Heart diseases**									
No		1			1			1	
Yes		2.97 (1.85;4.76)	0.000^***^		3.37 (1.61;7.04)	0.001^***^		2.76 (1.47;5.16)	0.001^***^
**Neuropsychiatric disorders**									
No		1			1			1	
Yes		2.91 (1.65;5.13)	0.000^***^		2.45 (1.11;5.39)	0.026^**^		3.79 (1.65;8.69)	0.002^***^
**Rheumatism**									
No		1			1			1	
Yes		0.26 (0.11;0.61)	0.002^***^		0.27(0.09;0.78)	0.015^**^		0.24 (0.06;1.02)	0.053^*^
**Other diseases**									
No		1			1			1	
Yes		8.94 (6.09;13.1)	0.000^***^		10.36 (5.75; 18,67)	0.000^***^		7.71 (4.61;12.87)	0.000^***^
**Intercept**	−13.80 (−16.42;−11.19)		0.000^***^	−13.81 (−17.72;−9.89)		0.000^***^	−13.32 (−16.80;−9.84)		0.000^***^
Correctly classified	85.05%			83.81%			86.17%		
Goodness of fit test^b^	0.0043			0.896			0.469		
Pseudo R^2^	0.307			0.328			0.308		

Notes: *** significant at 1%; ** significant at 5%; * significant at 10%.

Coeff.: Coefficient; OR: Odds Ratio; CI: Confidence interval.

a Odds ratios adjusted for wealth index, age, gender, history of hypertension, diabetes, heart disease, neuropsychiatric disorders, rheumatism and other chronic diseases (prior to stroke).

b P-value provided for Hosmer-Lemeshow test.

Analysis of lifestyle differences and risk factors among tertiles in the urban and rural population are presented in Table 4 (for qualitative variables) and Table 5 (for quantitative variables).

**Table 4:** Lifestyle differences among socioeconomic groups in urban and rural areas (test of proportions).

Qualitative variables	Urban population				Rural population			
	Proportions (%)			P-value^a^	Proportions (%)			P-value^a^
	Tertile1 (Poor)	Tertile2 (Interm.)	Tertile3 (Rich)		Tertile1 (Poor)	Tertile2 (Interm.)	Tertile3 (Rich)	
Smoking^b^	14.49	10.99	8.09	0.000	12.29	10.28	10.20	0.000
Practice of a sport^c^	11.83	16.82	24.29	0.000	2.25	4.80	8.44	0.000

Notes:

a χ^2^ test for comparing proportions between tertile 1 and tertile 3.

b smoker status obtained by interview.

c practice of a sport status obtained by interview, type and frequency of sport activity were not recorded.

**Table 5:** Lifestyle differences among socioeconomic groups in urban and rural areas (mean comparison test).

Quantitative variables	Urban population				Rural population			
	Mean value			P-value^a^	Mean value			P-value^a^
	Tertile1 (Poor)	Tertile2 (Interm.)	Tertile3 (Rich)		Tertile1 (Poor)	Tertile2(Interm.)	Tertile3 (Rich)	
Education (years)^b^	3.99	5.67	7.80	0.000	1.89	2.68	3.94	0.000
Nb of health consultations^c^	1.22	1.31	1.52	0.000	0.90	1.01	1.33	0.000
Frequency of red meat consumption^c^	2.67	2.32	2.15	0.000	2.68	2.56	2.44	0.000
Frequency of fruits and vegetables consumption^d^	1.14	1.10	1.09	0.000	1.13	1.11	1.09	0.000
Frequency of vegetable oil consumption^d^	1.08	1.09	1.12	0.000	1.05	1.06	1.06	0.063

Notes:

a t-test for comparing means between tertile 1 and tertile 3.

b Number of years of schooling successfully completed.

c Number of consultations over the last 12 months, obtained by interview. All types of providers were considered including medical and paramedical staff, and traditional healers (herbalist or fkih).

d Frequency of consumption obtained by interview. Figures range from 1 (every day) to 5 (never). The higher the value, the lower the frequency of consumption.

In urban areas, the proportion of smokers was significantly higher for the bottom tertile (T1 = 14.49%; T3 = 8.09%; p. value = 0.000) and the proportion of individuals having a leisure physical activity was significantly lower (T1 = 11.83%; T3 = 24.29%; P-value = 0.000). The average number of years of education and the number of medical consultations per year are both significantly lower in the bottom tertile as well. Regarding dietary habits, the consumption of red meat but also of fruits and vegetables (of which consumption reduces the risk for cardiovascular diseases) was less frequent in poorer households, while the consumption of vegetable oil was more frequent compared to richer households. The difference in consumption of other types of fat was not significant.

We obtained similar results for rural households although our analysis did not show a significant association between socioeconomic status and stroke prevalence in rural areas.

## Discussion

### Prevalence of stroke in Morocco

We found a crude prevalence of stroke of 284/100,000 for the population aged 15 years and older. The low crude prevalence rate could be partially explained by the age distribution of the study population. Prevalence and incidence of stroke increase dramatically with age and reach a peak in the elderly population. In our sample, the population most at risk of stroke (i.e. individuals aged 65 and over) represents only 7% of the overall population, which is relatively low compared to high-income countries. Yet, 65% of stroke survivors found in our study belonged to that age category, compared to 29% for individuals aged between 45 and 64 years and only 6% among those under 45.

As stated in previous publications on these data [25], the prevalence rate of stroke survivors in all ages extrapolated to the world's standard population is 292/100,000 (95%, IC 246-337), with no gender difference. The prevalence of stroke is higher in rural: 323/100,000 (95%, IC 270−445) than in urban areas: 282/100,000 (95%, IC 213−333), but the highest prevalence was encountered in peri-urban populations (491/100,000). Note that the term peri-urban refers here to a geographical area in the outskirts of a city; usually inhabited by new migrants from rural areas and characterized by a lack of infrastructure such as water, electricity, sewage, etc. In the rest of the article, the peri-urban population has been included in the urban population for the purpose of the analysis.

These results are in line with previous estimates of stroke prevalence for the WHO Eastern Mediterranean Region (EMR) which were used in the global burden of disease study in 2000. WHO Eastern Mediterranean region is divided in two sub-regions based on mortality strata: EMR-B is characterized by low child and adult mortality. EMR-D is characterized by high child and adult mortality and includes Afghanistan, Djibouti, Egypt, Iraq, Morocco, Pakistan, Somalia, Sudan, and Yemen. Truelsen et al. found a prevalence of respectively 320 and 368 per 100,000 for females and males in EMR-B subregion; and 294 and 292 for females and males of all ages in EMR-D subregion (which includes Morocco) [33].

Yet, these standardized prevalence rates remain relatively low when compared to high-income countries. A presumed high case fatality rate has been proposed by some authors as an explanation for the relatively low prevalence of stroke in developing countries [34]. The lower availability of emergency and general care for stroke patients in LMICs might result in a higher case fatality rate for both acute and chronic stroke patients. The resulting lower number of stroke survivors might be responsible for the lower stroke prevalence rate [35].

### Relation between SES and stroke and its social gradient

Several landmark literature reviews indicate that the socioeconomic status is intimately linked to the risk of stroke [7], . Our study in Morocco confirmed a significant association between socioeconomic status and prevalence of stroke (as measured by our wealth index). We found that the relationship is not linear and both the poorest and richest households have a lower prevalence of stroke compared to households with medium wealth level when we consider the overall study population.

Other variables such as hypertension, diabetes, pre-existing heart and neuropsychiatric disorders were associated with an increased prevalence of stroke. Interestingly, history of chronic rheumatism was negatively correlated with the prevalence of stroke. We believe that this is possibly due to the fact that these patients take chronically anti-inflammatory drugs i.e. aspirin and nonsteroidal anti-inflammatory drugs and their effect on systemic inflammation probably plays a role in reducing the incidence of stroke [36].

### Differences between urban and rural zones

Recent studies in developing countries found different levels of stroke incidence or related risk factors between urban and rural areas. High blood pressure is reported as the principal cause of stroke in Africa (with a population attributable fraction of about 51%) and several studies in African countries suggest that the prevalence of hypertension tends to be higher in urban than in rural areas [33]. In a study in mainland China, the relative risk for stroke among urban residents was 2.78 as compared to their rural counterparts [15]. A study on individuals aged over 65 years old in Latin America, China and India indicated that the crude prevalence of stroke varied considerably between sites, but was generally higher in urban than in rural areas [37]. Other studies in India have shown that risk factor levels identified in rural communities are still below those typically observed in urban parts of India [12], [38].

Although there are indisputable cultural differences between these populations, it is interesting to compare the situation of Morocco with other middle-income countries which are currently undergoing demographic and epidemiological transition. Interestingly, our analysis showed no statistical difference between rural and urban populations regarding the history of stroke. However, we observe that our wealth index somehow captures the area of residence of households since 80.52% of the bottom tertile are rural households while 95.65% of the top tertile are urban households. Indeed, these two variables are highly correlated (P-value = 0.00). This observation is corroborated by the fact that nearly 70% of poor people in Morocco live in rural areas. About 15% of the Moroccan population is considered as poor, with two-thirds living in rural areas, even if pockets of poverty exist in both urban and rural areas [22].

In fact, findings from other surveys in Morocco suggest that the level of stroke risk factors is higher in urban compared to rural populations. Benjelloun reported that hyper-cholesterolaemia, overweight and obesity were more prevalent among women, and in urban areas [16]. Diabetes affected men and women equally but was also more frequent in urban areas. The difference in anthropometric status between urban and rural populations may be explained by a difference in diet quality and lifestyle. Regarding diet, although total energy intake is higher in rural areas, it has a lower contribution from fats and from animal products. Regarding lifestyle, rural people have a higher caloric expenditure because of their agricultural occupation and transportation methods. The percentage of men smoking cigarettes was slightly higher in urban areas (34%) as compared to rural areas (30%). However, another study using data from the Moroccan national survey in 2000 indicated that hypertension was more frequent among rural residents [17].

So, although the area of residence was not significantly associated with the prevalence of stroke in our study, it appears that living in urban or rural area has an influence on the risk of stroke, but in an indirect way, as reflected by the socioeconomic status of urban and rural households.

### Stroke and related risk factors among urban residents

When differentiating households by their area of residence (urban/rural), we observed that the prevalence of stroke among urban households varies according to their wealth level, while there is no such evidence among the rural population. For urban households, we found that the OR for stroke among the bottom tertile was nearly twice as high as in the top tertile (see table 3). Our results show that the prevalence of stroke is higher among the most deprived individuals in urban areas.

In India, low SES (measured by education and income) has been associated with high rates of tobacco use and high prevalence of diabetes, particularly in urban areas [11], [39]. Risk factors for cardiovascular disease among more affluent urban Indian populations follow similar patterns to those observed in developed nations [12], [40]. Reddy et al. report a reversal of the social gradient (the inverse relationship of the prevalence of risk factors to the level of education) for hypertension, diabetes, tobacco use, and overweight in individuals from highly urbanized centers [14].

Taken together, these results suggest that the social gradient within the urban and most developed areas in emerging countries may be similar to HICs where high prevalence of many of the usual risk factors for stroke is found among the lower socioeconomic groups. The relationship between SES and stroke may however change at the country level when the overall population is considered. Looking at the case of Morocco, lower SES households from urban areas are generally wealthier than rural households. This could explain why the risk of stroke is higher for individuals with medium wealth level when we consider the overall population (both rural and urban households).

Several authors have studied this phenomenon and provide a possible explanation. Yusuf et al. noted that, with urbanization, there was a marked increase in consumption of energy rich foods and a decrease in energy expenditure because of less physical activity. Concomitantly, global influences (via television or increased availability of processed food) on lifestyles perceived to be desirable or modern are changing the types of food consumed in both urban and rural areas [32]. Yusuf et al. reported such changes in Korea, India, the Philippines, Indonesia and several other countries that have led to increasing rates of obesity, high blood pressure, cholesterol, and hyperglycemia, and a decrease in insulin sensitivity [32]. Ultimately, such changes can lead to an overall increase of stroke and cardio-vascular diseases (CVD) with higher rates encountered in urban areas. Urban, affluent, and educated sections of the population (early adopters) initially use their higher disposable incomes to experiment with risk-prone behaviors and therefore are at a greater risk of stroke and CVD. Later, as the mediators of risk (tobacco, unhealthy foods, and automated transport) become widely available for mass consumption, all social classes become affected. In the advanced phase of a CVD epidemic, the urban, educated and affluent sections of the population assimilate health information, adopt healthy behaviors, and access health care more efficiently [14]. These observations may explain why the probability of stroke may be higher in urban areas with an inversed social gradient showing a negative relationship between SES and stroke incidence.

Aidi et al. have reported a similar phenomenon in Morocco. Individuals from the poorest urban sections in Morocco mainly live in peri-urban areas and are composed of households who have recently moved from rural areas to live in slums at the outskirts of the main urban centers. Such peri-urban populations benefit from higher incomes than their rural counterparts, which lead them to adopt riskier behaviors in their new urban environment i.e. increased consumption of tobacco or adoption of a diet rich in fat and sugar, that they could not afford previously [25].

From these observations, we tried to isolate different behavioral patterns between the richest and poorest urban households that could explain differences in the prevalence of stroke. We observed that the level of education, the number of healthcare consultations and the number of persons having recreational exercise is significantly lower in poorer urban households while the number of smokers is significantly higher than in richer households. Findings regarding dietary habits are less conclusive as both healthy and harmful consumption patterns are observed in the poorer and richer households (i.e. the frequency of consumption of red meat but also of fruit and vegetables is higher for richer households). Nevertheless, our results suggest for the most part that the higher prevalence of stroke in the poorest urban households could be explained by a higher presence of recognized risk factors in this part of the population.

### Implications for stroke prevention in Morocco

Cardiovascular disease and stroke are rapidly growing problems in LMICs, and are major causes of illness and deaths in WHO EMR, accounting for 31% of total deaths. Approximately 75% of cardiovascular disease can be attributed to conventional risk factors [41]. Stroke can be prevented with sound information and better prevention strategies; but improved surveillance, prevention and control of hypertension and stroke in the Middle East and North Africa are much needed to lower the prevalence of stroke in that region [42]. Primary prevention strategies should cover both population-wide and individual level interventions aimed at reducing the risk of cerebrovascular disease for example through the voluntary or regulated reduction in dietary salt intake, control of blood pressure and hypercholesterolemia, and combination drug therapy for individuals at high risk of a cardiovascular event [43].

In Morocco, the *Programme National de Prévention et Lutte contre les Maladies Cardiovasculaires* [National Program for the Prevention and Control of Cardiovascular Disease] has focused on hypertension and rheumatic heart disease during the period 2008−2012. For BP, the objective is to reduce the burden of hypertension through primary prevention (by promoting healthy lifestyles) and a more effective management of hypertension. With regard to rheumatic heart disease, the objective is to reduce the incidence and prevalence of the disease [44].

It is widely agreed that the combination of population-based and individual prevention strategies are complementary strategies that provide a continuum of interventions and offer the best chance for effective stroke prevention [41], [45]. Specific, evidence based information about exposure to stroke risk factors and stroke prevalence can help developing appropriate health promotion strategies. According to our results, targeting stroke prevention efforts on poor urban households can maximize the potential for primary prevention activities and participate to the effective implementation of stroke prevention. We hope that the results presented in this study will help to guide the design and setup of the national program for the prevention and control of cardiovascular diseases in Morocco.

### Strengths and limitations of the study

Our study is based on a rigorous epidemiological survey of stroke and stroke-related risk factors in Morocco; which used standard definitions and tools to diagnose and classify stroke; and collected data from a large representative sample of both urban and rural populations; with a good specificity and sensibility of the questionnaire in both settings. In addition, the use of a statistically weighted, asset-based index is considered as a robust method to produce welfare rankings of the population; and this approach is widely used - particularly in LMICs - where accurate expenditures or income data may be difficult to collect [27].

Yet, some limitations of the study should be considered when interpreting our findings. Firstly, some restrictions arising from the data collection process may explain why some common risk factors do not appear significantly associated with stroke when they are introduced in our model. For example, with regard to cigarette smoking, we only considered if a person was smoking or not at the time of the survey, although the number of years of tobacco consumption or the quantity of cigarettes smoked per day do have an influence on the risk of stroke [46]. The effect of tobacco consumption may also be underestimated, given that our control group (non-smokers) includes individuals who have quit smoking or who are exposed to passive smoking. Also, regarding physical activity, we only considered recreational exercise (sport) that is practiced more rarely in poorer households but we do not take into account physical activity related to work or transport. We had no information either on the frequency or the type of sport and hence did not have information on the total caloric expenditure. Moreover, for dietary habits, we only considered the frequency of consumption of various types of food and did not have any information on the quantities consumed or calories taken in. At last, we could not investigate the association between stroke and some potential risk factors such as alcohol consumption or Body Mass Index (BMI) due to lack of data.

In addition, household socioeconomic status was assessed at the time of the survey and we did not have information on the situation prevailing before the stroke attack. However, we know that for stroke survivors, the duration between the attack and the interview was 44 months on average. Thus the risk of bias is somewhat mitigated by the fact that we used a wealth index based on dwelling characteristics and ownership of assets as a proxy for households' socioeconomic status. Such measure is representative of the long term income of households and thus less prone to short term fluctuations; which is an advantage compared to other measures of SES based on households' income or expenditure per capita.

Finally, our sample is representative of the general population in Morocco in terms of age, gender and the urban/rural distribution. However, the survey was not designed to capture the ethnic background of respondents (e.g. Arab or Berber) which may well have an influence on lifestyles; this may limit the transposition of our results to specific ethnic groups in Morocco.

In light of the above limitations, further research could be conducted to better understand the behavioral and lifestyle differences that could explain variations in stroke prevalence among socioeconomic groups in Morocco. In addition, it would be useful to explore if there are any significant variations in individuals with diverse ethnic backgrounds. We also suggest that additional studies are carried out to investigate the association between SES and stroke prevalence using other proxies of socioeconomic status such as (adjusted) household expenditure or income, education, occupation, etc.

## Conclusion

The link between SES and stroke applies to countries at different levels of their development; however the nature of this relation changes according to the advancement in their demographic, economic and epidemiological transition. Findings from various studies suggest that the link between SES and stroke at the country level may occult different realities between urban and rural populations.

Emerging patterns in developing countries should continue to be monitored to maximize the potential for primary stroke prevention activities by focusing on the part of the population enduring the highest risk. In this study, we found a significant association between household socioeconomic status and prevalence of stroke. Our main result is that individuals from the most deprived urban households bear a higher probability of stroke compared to the rest of the population in Morocco. This result could be explained by the higher presence of behavioral risk factors (unhealthy diet, insufficient physical activity, tobacco smoking, etc.) in this specific category of the population which leads in turn to metabolic/physiological risk factors of stroke such as diabetes, hypertension, obesity, etc.

Hence, it is important that policy makers focus their efforts on primary stroke prevention. In addition to health education and policies aimed at facilitating the adoption of healthy lifestyles by individuals, targeting stroke prevention efforts on poor urban households can participate in the effective implementation of stroke prevention to tackle the growing related burden in Morocco.

## Supporting Information

Table S1 **Value labels, codes and frequencies of each modality of the 15 variables considered for the MCA.**
(DOCX)
