# Supplementary material for: Socioeconomic Status and Stroke Prevalence in Morocco: Results from the Rabat-Casablanca Study
Source: PLoS One. 2014 Feb 28;9(2):e89271. doi: 10.1371/journal.pone.0089271 (PMC3938460; doi:10.1371/journal.pone.0089271)
Supplement: Table S1 — Value labels, codes and frequencies of each modality of the 15 variables considered for the MCA. (DOCX) [file pone.0089271.s001.docx]

Table S1. Value labels, codes and frequencies of each modality of the 15 variables considered for the MCA.

| **Dwelling characteristics** | **Categories** | **Number** | **Proportion (%)** |
| --- | --- | --- | --- |
| **Type of habitation** | Detached house or equivalent | 1,334 | 2,22% |
|  | Flat | 9,876 | 16,45% |
|  | Traditional dwelling | 22,059 | 36,75% |
|  | Informal dwelling/shack | 11,060 | 18,43% |
|  | Rural dwelling/solid wall | 9,015 | 15,02% |
|  | Rural dwelling/wattle and daub | 5,582 | 9,30% |
|  | Others | 1,093 | 1,82% |
|  | Missing values | 12 | - |
| **Number of rooms** | 1 | 5,182 | 8,63% |
|  | 2 | 17,814 | 29,67% |
|  | 3 | 21,569 | 35,93% |
|  | 4 | 9,129 | 15,21% |
|  | ≥5 | 6,337 | 10,56% |
| **Source of water supply** | Piped (tap) water | 39,329 | 65,59% |
|  | Public/communal tap | 8,405 | 14,02% |
|  | Spring water | 1710 | 2,85% |
|  | Borehole | 10,403 | 17,35% |
|  | Others | 117 | 0,20% |
|  | Missing values | 67 | - |
| **Lighting** | Electricity from mains | 48,287 | 80.53% |
|  | Electricity from generator | 363 | 0,61% |
|  | Candles | 1,821 | 3,04% |
|  | Gas | 6,735 | 11,23% |
|  | Others | 2,755 | 4,59% |
|  | Missing values | 70 | - |
| **Kitchen** | Exclusive | 57,136 | 95,21% |
|  | Shared | 1,553 | 2,59% |
|  | No facility | 1,322 | 2,20% |
|  | Missing values | 20 | - |
| **Toilet facility** | Exclusive | 51,800 | 86,32% |
|  | Shared | 2,762 | 4,60% |
|  | No facility | 5,450 | 9,08% |
|  | Missing values | 19 | - |
| **Bathroom** | Exclusive | 28,174 | 46.95% |
|  | Shared | 871 | 1.45% |
|  | No facility | 30,966 | 51.60% |
|  | Missing values | 20 | - |
| **Television** | Yes | 54,882 | 91,43% |
|  | No | 5,141 | 8,57% |
|  | Missing value | 8 | - |
| **Satellite dish** | Yes | 39,510 | 65,82% |
|  | No | 20,513 | 34,18% |
|  | Missing value | 8 | - |
| **Phone** | Yes | 10,410 | 17,34% |
|  | No | 49,606 | 82,64% |
|  | Missing value | 8 | - |
| **Cell phone** | Yes | 54,347 | 90,54% |
|  | No | 5,676 | 9,46% |
|  | Missing value | 8 | - |
| **Stove** | Yes | 47,174 | 78,59% |
|  | No | 12,849 | 21,41% |
|  | Missing value | 8 |  |
| **Fridge** | Yes | 43,139 | 71,87% |
|  | No | 16,884 | 28,13% |
|  | Missing value | 8 | - |
| **Washing machine** | Yes | 27,223 | 45.35% |
|  | No | 32,800 | 54.65% |
|  | Missing value | 8 | - |
| **Car** | Yes | 13,498 | 22,49% |
|  | No | 46,523 | 77,51% |
|  | Missing value | 10 | - |
